# ‘The book’s a conversation starter’: a realist exploration of the salutogenic potential of reading for pleasure

**DOI:** 10.1136/medhum-2023-012880

**Published:** 2024-04-30

**Authors:** Mihirini Sirisena, Monique Lhussier, Eileen Kaner, Angela Wearn, Joanne Gray, Rebecca James, Sam Redgate

**Affiliations:** 1 Population Health Sciences Institute, Newcastle University, Newcastle upon Tyne, UK; 2 Department of Social Work, Education and Community Wellbeing, Northumbria University, Newcastle upon Tyne, UK; 3 Office for Health Improvement and Disparities, London, UK

**Keywords:** arts in health/arts and health, Qualitative Research, Mental health care

## Abstract

Reading for Wellbeing (RfW) is a pilot initiative, aimed at improving mental health and well-being through supporting access and increasing opportunities to read for pleasure. RfW was implemented across six North-East local authorities in England and employed Community Reading Workers to support access to books and reading for targeted populations. The current study used realist methodology to understand context, potential mechanisms of action, acceptability and reported outcomes. Data generation and analysis were conducted iteratively, using focus groups, interviews and observations.

The analysis of the collated data highlighted that a positive attitude towards reading and a desire for social connections were significant motivators for engagement with RfW. This paper postulates eight programme theories relating to that context, which describe key mechanisms within RfW linked to engagement with reading, well-being, connections and practice. The paper concludes that previous notions of positivity associated with reading for pleasure enable participants to experience RfW as a positive social encounter. This positive social encounter enhances participants’ multiple resistance resources such as increased sense of self-efficacy and connectedness that could impact on their sense of well-being.

## Introduction

 Reading for Wellbeing (RfW) was a pilot initiative in which Community Reading Workers employed by Local Authorities supported targeted populations in socioeconomically deprived regions of North-East England by enabling access to books and reading with a view to enhance mental health and well-being . The project was initiated and part-funded by author Ann Cleeves, to mark the twenty-first anniversary of her detective character Vera Stanhope, and in recognition of the solace she found in stories throughout her life. A key assumption underlying the project was that opportunities for reading for pleasure could mitigate the effects of hardships facing disadvantaged communities. Conceptualisation of RfW was founded on growing evidence presented in a number of studies, which have found positive links between reading and health (Davis *et al* 2016), particularly mental well-being (Billington *et al* 2013). These studies have noted that the ability to represent reality (Black and Barnes 2015) and relatability (Malyn, Thomas, and Ramsey‐Wade 2020) in texts read for pleasure facilitate an immersive experience (Bavishi, Slade, and Levy 2016; Billington, Davis, and Farrington 2013), which can affect readers’ sense of well-being. Reading exposes readers to other people’s lives and worlds (Albjerg 1962; Longden *et al* 2015); enabling them to feel diverse emotions without experiencing consequences of associated actions (Gray *et al* 2016); facilitating the potential to turn reading into a mimetic experience (Canty 2017; Lanza 1996; Fuller and Procter 2009) and form emotional connections (Bal and Veltkamp 2013; Brewster and McNicol 2021; Thumala Olave 2018). RfW assumed that outcomes engendered through reading for pleasure enhance salutogenic assets—resources that individuals and communities have or could access, which would protect against negative health outcomes and enhance positive health (Morgan and Ziglio 2007). The project postulated that enhanced salutogenic assets would enable people living in disadvantaged communities to manage various stressors encountered in everyday life.

Pioneered by Aaron Antonovsky, the theory of salutogenesis, which views health as a continuum from ease to disease, has been gaining traction in the field of health promotion in recent years (Mittelmark *et al* 2017; Morgan, Davies, and Ziglio 2010). Antonovsky proposed that a strong sense of coherence—a disposition that helps one to perceive life as comprehensible, manageable and meaningful—enables people to move towards ‘the ease’ end of the health spectrum as it increases their capacity to face diverse stresses while maintaining a status of good health (Antonovsky 1996). In his view, what facilitates a greater sense of coherence is people’s access and ability to mobilise ‘resistance resources’—generalised and specific, and internal and external capacities, capabilities and relationships—that equip people to manage their encounters with various stressors (Mittelmark and Bauer 2017; Tan, Vehviläinen-Julkunen, and Chan 2014; Huss and Samson 2018). Approaching salutogenesis as an orientation to life, some argue that sense of coherence is one of many salutogenic assets that could enhance a person’s sense of well-being, which sit alongside assets such as resilience, connectedness, self-efficacy, cultural capital and learnt hopefulness (Eriksson and Lindstrom 2010). Mobilising salutogenic assets within health promotion interventions could strengthen a person’s sense of coherence, which could lead to increased sense of self-acceptance, tolerance of a variety of emotions, perceptions of quality social support, and coping abilities promotes mental health (Langeland *et al* 2007; Langleland and Vinje 2022). It has been noted that participation in creative activities, such as reading, engage cognition, emotion and senses, which could impact on perceptions of life as manageable, comprehensible and meaningful (Huss and Samson 2018), as well as enhancing salutogenic assets at the level of the individual (Jensen 2019).

This paper presents the findings of a realist evaluation of the RfW project, which aimed to understand contexts, potential mechanisms of action, acceptability and outcomes of the initiative.

## Methods

### The intervention

The RfW project was a multiagency co-funded pilot scheme, which involved the appointment of nine ‘Community Reading Workers’ across six local authority areas, experiencing diverse contexts such as population density, ethnic diversity and rural/urban geographies. The project idea was presented by Ann Cleeves at a public meeting in 2020, who committed personal funds to the project on the condition that it was match-funded and that the initiative be independently evaluated. Ann Cleeves continued to play a key role in the design and implementation of the project. The project was implemented in areas where populations were experiencing high levels of socioeconomic deprivation, and where there was an existing enabling infrastructure including relationships with key partners in public health, library services, community hubs and social prescribing link workers. The project was implemented from June 2021, as a 1 year pilot project in the first instance.

The overarching objective of RfW was to increase access and opportunity for reading for pleasure by addressing barriers to reading for pleasure. The project embraced an ethos of reader centredness and a broader understanding of reading. Within this approach, prominence was given to the content that mattered to the reader and a wide range of formats were understood to be acceptable forms of reading, which ranged from comics, magazines, audiobooks to serious literature. Community Reading Workers attended a bespoke training programme on implementing these principles. The operationalisation of the project was informed and guided by local need and community assets available in the locality. Across all pilot areas, Community Reading Workers either introduced the project to established groups who met in community settings or founded new groups for which participants were recruited via advertising on social media, community networks, social prescribing networks and Local Authority networks. Key characteristics shared across sites included the Community Reading Workers arranging group meetings at an agreed place and time and facilitating conversations around what participants had read or are reading. Community Reading Workers often took a selection of books to the meetings, which included books requested by participants.

The evaluation research developed alongside RfW, with input from key stakeholders and a team of academic advisors. The conversations with stakeholders and academic advisors informed development of initial programme theories (IPTs), which guided the evaluation.

### Study design and ethics

We used a realist evaluation approach to understand the context of implementation, potential mechanisms of action, acceptability, and explore perceived outcomes of RfW to those delivering and experiencing RfW. Within realist evaluations, interventions (such as RfW) are viewed to operate through introducing new resources into existing social relationships, thus creating mechanisms for change by modifying capacities, constraints and choices for participants and practitioners (Judge 2000). Taking this approach allowed for causal explanations to be developed highlighting how RfW was able to impact on a number of outcomes for participants, with particular reference to the role of context, rather than simply asking ‘did it work?’. Realist approaches emphasise that interventions such as RfW only ‘work’ through the choices, intentions and behaviours of participants as they engage with the resources provided by the initiative (Duncan *et al* 2018). Thus, it is well suited to studying complex social interventions such as RfW with the potential for multiple pathways from implementation to impact.

Realist evaluation attends to the ways that interventions may have different effects for different people, by trying to understand configurations of contexts and mechanisms that link to outcomes (C+M=O). The mechanisms are further broken down between the resources brought by the intervention and the response (or reasoning) that participants have as a result, which leads to observable outcomes (Dalkin *et al* 2015). CMO configurations are referred to as programme theories; the ideas and assumptions underlying how, why and in what circumstances complex social interventions work and they are the units of analysis used within realist evaluation (Best *et al* 2012).

IPTs are first developed, refined and then tested through the research, leading to the formulation of refined programme theories. This framing allowed for the generation of rich, causal explanations to convey ideas and assumptions underlying how, why and what circumstances RfW might work to achieve the intended effects. This results in contextually dependent theories which are specific enough to enable testable propositions, and sufficiently generalisable, to apply in different settings in order to allow for this learning to benefit other similar interventions.

Informed consent was obtained from all participants at the start of the research. Interviews and focus group sessions were recorded with participant consent, which were anonymised prior to transcription.

### Patient and public involvement statement

The public were involved in the design and analysis plans of our research. Table 1 outlines how public engagement activities informed the evaluation.

**Table 1:** Public engagement activities

Event	When	Who was involved	The focus	Action taken
PICE consultation 1	October 2021—prior to data generation	Practitioners (n=7) and public members (n=3)	To assess and refine the outcome questionnaire	Updated the outcome questionnaire
PICE consultation 2	July 2022—midpoint of data generation	Public members (n=4)	Sharing early findings from outcome and process evaluations for sense-checking	Topic guide was refined to address the comments and feedback shared
PICE consultation 3	April to May 2023—following the analysis of data generated	Public members at community centres (n=18)	Sense-checking and refining programme theories/findings	Informed the refinement of programme theories

PICEpublic involvement and community engagement

### Data generation

Data for the research were generated through a multimethod approach between July 2021 to November 2022 and was conducted in three stages (see table 2 for detail on data generation and table 3 for information on practitioners who were involved in the research). Stage 1 focused on understanding the development and implementation of the project, which provided a framework for the research. This helped us to develop IPTs and inform topic guides for interviews and focus groups in the subsequent stages. Stage 2 comprised of observations of project delivery and focus groups and interviews with project participants, identified with the support of community reading workers. The generated data tested and refined the IPTs. The early findings were shared with a small group of experts by experience, whose feedback and comments informed subsequent data collection and analysis. Stage 3 included further interviews and focus groups with participants with a revised topic guide. Analysed data were shared with a second group of experts by experience comprising of non-participant members from communities where RfW had been implemented in a process of theory refinement (Manzano 2016). Eight programme theories were identified following this public involvement activity.

**Table 2:** Overview of qualitative data collection

Stage 1	Interviews with practitioners	Local leads	8 individuals, 6 interviews
		Reading project workers	9 individuals, 6 interviews
	Observations	Local Steering Group meetings	26 (approximately 26 hours)
		Regional Steering Group meetings	6 (approximately 9 hours)
	Informal conversations with Reading Workers	Number of practitioners	7
		Number of occasions	3 (approximately 3 hours)
Stage 2	Data collection from participants	Observations at project delivery sessions	4 (approximately 6 hours)
		Focus groups with participants	3 (n=17)
		One-to-one interview	1
Sharing early findings with PICE members			
Stage 3	Data collection from participants	Focus groups with participants	9 (n=28)
		One-to-one interviews with participants	3
Sharing early findings with a group of experts by experience			

PICEPublic Involvement and Community Engagement

**Table 3:** An overview of practitioners involved in the evaluation research

Practitioners	Local leads	Eight individuals who were involved in the implementation of RfW, employed by Local Authorities, who were based in departments affiliated with library services, cultural services and public health
	Reading project workers	Nine individuals employed (two full-time and seven part-time) or seconded by the Local Authorities, followed a training to become Community Reading Workers and were responsible for delivering RfW (participant recruitment, designing and delivering RfW activities)
	Local steering groups	Local steering groups comprised of local healthcare trust representatives, VCS and statutory organisations involved in social prescribing and early years provisions
	Regional steering group	Regional steering group comprised leads at local authorities, VONNE and OHID officials, Ann Cleeves

OHIDOffice for Health and Improvement DisparitiesRfWReading for WellbeingVCSVoluntary and Community SectorVONNEVoluntary Organisations’ Network North East

### Analysis

Data generation and analysis were conducted iteratively. The analysis was embedded in NVivo, which enabled a grounded and iterative approach (Dalkin *et al* 2021). As inherent in realist approaches, the analysis sought to find patterns in the data as well as identify counteracting threads, when they were present. 12 IPTs were identified following interviews with local leads and project workers at the beginning of the research (Stage 1). These IPTs were used as nodes to organise and analyse data generated through observations at project delivery sessions and focus groups and interviews with participants (Stages 2 and 3).

## Findings

A positive attitude towards reading for pleasure emerged as the key dynamic affecting the uptake of the RfW offer. Participants who engaged with the project described themselves as ‘avid readers’ indicating they read frequently, or readers who have ‘lapsed’ or ‘fallen out of the habit of reading’. Participants conflated reading with enjoying stories in book format, including printed and audiobooks.^1^ This was contrasted against consuming stories in visual formats as in film or tele series. In addition, a desire to make reading a social experience appeared to motivate participants to engage with RfW. Findings clustered around four themes: enhancing enjoyment in reading, well-being, connectedness and practice. Within these themes, eight programme theories offered explanations for how RfW was viewed to work, for whom and in which circumstances . They are presented below.

### Theme 1: Enhancing enjoyment in reading

The project’s aim was to increase access to reading for pleasure. The concept of access was interpreted within the project in a multifaceted way, which included addressing material and psychological barriers facing participants as well as expanding the opportunity for enjoyment through broadening choice. Findings indicated that Community Reading Workers offered avid and lapsed readers resources that enhanced their reading habits.

#### Workers mobilised skills and knowledge to address barriers to reading for pleasure

For ‘lapsed’ readers, Community Reading Workers appeared to mobilise their community working skills and knowledge of reading resources to address stressors and facilitate access to reading for pleasure. In the following paragraphs, we detail key barriers participants experienced and solutions made available by Community Reading Workers.

##### Audiobooks

Physiological changes, such as deteriorating eyesight and arthritis were highlighted as barriers to reading by ‘lapsed’ readers. In addition, conditions such as Dyslexia were also mentioned to make reading challenging. Community Reading Workers introduced audiobooks to these participants and raised their awareness of resources such as Borrowbox, an audiobook lending scheme which can be accessed via library services. Participants explained that the introduction to audiobooks enabled them to access reading for pleasure and find relaxation.

With my dyslexia, I'm more of an audiobook person because I can verbally do more than what I can when I'm looking at things…. Sometimes I can't physically pick up a book, so I do like to listen to a book, and I can just melt away.

(Focus group, Area 4a)

While some participants found audiobooks to be an effective way to enjoy reading, some others indicated that audiobooks did not facilitate the same escapism as reading a book because dynamics such as the voice or the tone in which a book is read interfered with their potential for enjoyment.

###### Quick-reads

Being introduced to ‘quick-reads’ rekindled the interest in reading for pleasure for ‘lapsed’ readers. Quick-reads are a format of books that are short, easy to follow and are printed in large print. Participants explained that these require less mental energy and could fit around their daily tasks. They added that getting back into the habit of reading for pleasure with quick-reads enabled them to ‘steal’ moments of relaxation and catharsis amidst everyday busy-ness.

And I mean, you pick all these books up and they're like this big, and I'm like, “I just haven't, how am I going to get through this? I can't see us ever getting to the end of this.” … But then when she [community reading worker] bought the quick-reads in, I just kind of read the first couple of chapters and before I knew it, I was basically at the end, and I was like, “You know what, I've literally read this in two hours.”

(Focus group, Area 4a)

###### Finding time

For participants struggling to find time to read, workers suggested strategies such as reading instead of reaching out to social media or listening to an audiobook while doing household tasks or driving. These strategies enabled participants to interject reading into everyday life easily, leading them to finding enjoyment in reading.

Instead of picking my phone up I was picking up a book. But then I was going to sleep a lot easier. Instead of being on my phone until … I'd be asleep at a reasonable time….

(Focus group, Area 5a)

Overall, workers with interpersonal skills, knowledge of different formats of reading and ways of accessing stories appeared able to mobilise a combination of strategies to support ‘lapsed’ readers to consider that reading could be enjoyed in different formats and could be incorporated around other activities and responsibilities. This led to the formation of programme theory 1:

For readers who have lapsed due to physiological and psychological barriers to reading for pleasure (Context), Community Reading Workers being able to make effective recommendations (resource) enable the lapsed readers to consider reading to include a wide variety of formats (reasoning) leading to finding enjoyment in reading through escapism and relaxation (outcome).

### Awareness of different genres and authors enhances reading for pleasure

RfW participants found that engaging with the project enhanced their reading experience through broadening awareness of books available. They pointed out that conversations with workers and fellow participants, workers bringing diverse books to group meetings and holding events such as author visits, roused their curiosity and motivated them to explore other genres and expand their choice, leading to discovering new ways to find enjoyment in reading.

You tend to just think, “Oh, well, I like that one, so I'll read that one- you know, something by her again.” But if somebody says to you, “Oh, try that one.” And you may read them and think, "Well, I would never have read that in a million years. I would have looked at the start, but everyone else has said it’s really good, so come on, plough on with it, you know?” And it does open your mind a little bit more, you know?

(Focus group, Area 2)

Individual participants elaborated how this mechanism influenced them in distinctive ways. Exposure to different books enabled those who did not feel confident to venture out into books/genres they were not familiar with as well as those who felt that familiarity with storylines had begun to wane their enthusiasm for reading. One participant felt being exposed to new genres encouraged her to make new choices and decisions about reading, which increased her confidence in making decisions about other aspects of life such as accessing new services.

One participant hypothesised that just as expanding reading choice, bringing reading-related resources to groups where participants might not share a reader identity may motivate them to read for pleasure. He elaborated that in the group he attended, a worker bringing books related to a popular topic of interest incited the groups’ enthusiasm to read.

Programme theory 2 postulates:

For people who read for pleasure (context), exposure to new genres and authors in RfW groups (resource) motivates them to explore new books (reasoning) leading to finding enjoyment in reading in new ways (outcome).

### Theme 2: Well-being

Participants reported several RfW well-being-related outcomes, connected to the act of reading for pleasure and meeting as groups. Participation in the project was shown to encourage participants to proactively consider well-being and mental health. This was particularly the case for those who were facing emotional hardship due to life-changing circumstances, such as bereavement, retirement, illness, care responsibilities or moving into new neighbourhoods.

#### Reading as an act of self-care

Participants reported that discussions within RfW groups encouraged them to consider the potential impact of reading for pleasure on their own well-being and helped them to challenge previously held views, such as believing that reading is a selfish act and feelings of guilt for spending time reading for pleasure rather than undertaking other tasks. The project appeared to create a space that gave participants permission to *be* themselves, focusing on their interests while shelving their other roles and responsibilities, although for a short moment in time. This appeared to facilitate the thinking that this is part of self-care. Participants reported that practising self-care by embracing an activity they liked lead to an increased sense of well-being.

It’s that self-care isn't it? To sit and read is taking time away from the laundry or time away from the children or time away from doing all these other things that are taking the time of your life, which actually finding that time for yourself is a massive benefit.

(Female, Focus group, Area 5b)

However, one participant shared in an interview that explicit associations with well-being and mental health may put off participants joining RfW due to perceptions of stigma attached to mental health.

Programme theory 3 suggests:

For participants who consider reading as a hobby (context), discussions in RfW groups about reading and well-being (resource) enable them to consider taking time for reading for pleasure as self-care (reasoning) leading to improved well-being (outcome).

#### Reading for well-being gives a sense of purpose

An overarching reflection of participants was that the project was a planned activity that was scheduled to take place at a set time and date and a specific venue. This enabled them to arrange their day, week or month around it, which was particularly important for participants who were experiencing life-changing events, or those who felt that they did not have a reason to leave the house. While a majority of participants were motivated by an interest in reading, one participant shared she decided to join RfW, despite not having an interest in reading, as she was looking for activities as motivation to leave the house. Having a planned activity to attend appeared to provide a sense of purpose, such as a reason to leave the house. Further, having an activity around which they could plan their day appeared to interject a sense of structure to their day. Participants reported that having a sense of purpose led them to feel an increased sense of self-efficacy, through enabling them to have an increased a sense of confidence in their ability to manage life.

That’s part of this wellbeing as well, because I'm going to [place] or whatever I'm doing, I'm going to [town], so even today I've had my lunch, I've had my breakfast, I've had my cup of tea before I've joined you,… All of a sudden you start looking after yourself without realising it… if I've got to be somewhere I've got to make sure that I've got nutrition, I've got to have something to eat.

(Interview 2, Area 2)

Programme theory 4 indicates:

For participants who like to read, particularly those experiencing life-changing events or those who feel that they do not have a reason to leave the house (context), having a planned reading activity taking place at a set date, time and venue (resource) provides an anchor around which to build everyday activities (reasoning) leading to an increased sense of self-efficacy (outcome).

### Theme 3: Connectedness

Implementing reading for pleasure activities in group settings facilitated pathways for social connections that were further enhanced through association with reading. These pathways emanate from a shared interest in reading, which enables participants, particularly those who were less likely to join other interest-based groups, to establish a commonality through which familiarity and trust appeared to emerge.

#### RfW groups facilitate companionship

Participants highlighted companionship as a valuable experience facilitated through RfW groups. This was particularly valued by participants who described themselves as introverts who liked reading and participants who were facing emotional hardship such as feelings of loneliness, lack of purpose or feeling overwhelmed, which were linked to life-changing events, care responsibilities or lifelong ill mental health. Participants explained that sharing an interest and value in reading enabled them to find a common ground with others, which appeared to initiate a network of connections. Further, participants shared that regularity of meetings combined with not feeling pressured to participate in any defined way enabled them to feel a sense of acceptance and experience a sense of belonging in a meaningful way as the contact is not fleeting. It was reported that the combination of these dynamics enabled participants to find a space for relaxation and recuperation, leading to a sense of increased well-being.

That can be a wellbeing thing, just hearing other people talking. You don't necessarily have to be the one that talks. You can be the one that listens. Again, that’s where you feel comfortable.

(Interview 2, Area 2)

Programme theory 5 postulates:

For readers who are introvert or those experiencing emotional hardship (context), RfW provides an opportunity to meet other readers with whom they share an interest (resource) enabling them to feel a sense of companionship (reasoning) leading to a sense of connectedness (outcome).

#### Books offer limitless scope for discussion

Participants pointed out that scope for discussions in RfW groups offered the potential to explore a multitude of topics and interpretations. In addition, the opportunity to relate stories to participants’ personal experiences enhanced the richness of the discussions. It was shared that these explorations enabled participants to consider the reading as well as their experiences from different perspectives, deepening their understanding and the enjoyment of reading as well as their understanding of self and the world. Some participants made a distinction between listening and speaking as leading towards different outcomes. They suggested that listening to other perspectives enabled them to broaden theirs. While the opportunity to speak and share opinions enabled them to feel that they were heard and their viewpoints valued and validated, leading to feelings of increased sense of self-worth. Both pathways appeared to lead to an increased sense of empathy, trust and respect towards other group members, resulting in strong connections.

I think with reading, because it is so varied, you cover so many topics and a lot of the time, we do not talk about the books, but we move onto other things. Whereas, if you are doing a group maybe with art or something, it is about art all the time. Whereas this, you cover so many different subjects. It is all encompassing really, isn't it?

(Focus Group, Area 3)

Participants of a pre-existing group where RfW had been introduced shared that discussions about books provided a new way to connect with each other. Discussions enabled them to explore themes and points of view which hitherto had not been explored, which the participants felt, enabled them to get to know each other in different ways.

Further, discussions appeared to present a learning opportunity for Community Reading Workers to challenge their viewpoints relating to the implementation of RfW. Discussions alerted the workers to different ways readers are affected by and relate to books, which challenged their preconceptions, and enabled them to make more appropriate recommendations.

Participant: … I think sometimes you need to have a book that you can emotionally attach to rather than just having a holiday romance or something. It’s good for you to kind of get those emotions out and to feel. Variety. … Then you feel like you've got a better way of dealing with it if you come across some of those things similar in the future.

Worker: … I've just thought, I've never thought of this other than when you were just speaking then, I wonder then if it’s like a safe way to feel emotion. ….

(Focus Group, Area 5b)

This Community Reading Worker had previously shared that broadening choice was particularly important in the locality she was working in, where readers repeatedly turn to books with distressful content. However, readers from that area reported that such books offer an opportunity for catharsis and the distance between the book and oneself offers a safe space to process emotions, particularly those such as fear, anger, disappointment.

Programme theory 6 proposes:

For people who come to RfW groups (context), the opportunity to explore diverse viewpoints and experiences through the discussion of stories (resource) enable group members to broaden their understanding of their lives and the world (reasoning) leading to being able to connect better with each other (outcome).

### Theme 4: Practice development

#### Community Reading Workers put participants at ease

A recurrent theme participants highlighted in interviews and focus groups was the key role the workers played in creating facilitatory conditions within RfW. Community Reading Workers’ demeanour such as being calm, observant, non-intrusive, attentive, willing to listen combined with their knowledge, experiences, and passion for reading provided them with a pool of resources, which they mobilised to engage with participants effectively. Participants described that through understanding and addressing their diverse needs and capacities, the workers create an inclusive environment where a broad range of people felt welcome. In groups specifically formed for RfW, it was noted that Community Reading Workers were required to play a facilitatory role in creating an environment where participants felt at ease about participation. Some of these groups highlighted that continued involvement of the worker is a key requirement for the group to continue. For more anxious participants, the workers provided further assistance, such as meeting prior to the session on a one-to-one basis, to enable them to feel at ease about participation. For pre-existing groups to which RfW was introduced, the workers adopting a perceptive and sensitive role so as not to affect the group dynamics facilitated engagement with reading. Across these settings, participants shared that the workers’ commitment to the project—their knowledge of books, commitment to reading, commitment to catering to their needs—enhanced participants’ motivation to read and participate in RfW.

…[worker] has come into that [group] so smoothly and interacted with us in a way that is so pleasant and positive. And reacted so attentively to what we have said. Whatever we have said, she has picked up those little nuances of what we might be interested in, and then thought around that - “Okay, if this person is interested in this author, they might be interested in these.” It’s that sort of level of interaction, the quality of interaction, and attentiveness.

(Focus Group, Area 1b)

Programme theory 7 suggests:

When delivering RfW project (context), Community Reading Workers activating their personality, interpersonal skills and reading-related resources (resource) enable them to create an environment where participants are made to feel at ease (reasoning) leading to more participants meaningfully participating in the project (outcome).

#### Project delivery environment influences ideas about accessibility

Participants shared that the qualities of the space where the project activities were delivered influenced their thoughts about how accessible the project was. Transport links, size of the room, acoustics of the space, and familiarity with the space influenced people’s willingness and curiosity to participate. In addition, dynamics such as activities being free and the project location being an accepting space where the participants felt they did not have to be concerned about appearances, helped them view the project as accessible.

That helps your mental wellbeing as well doesn't it? Coming somewhere you don't have to worry that the kids might be misbehaving. You don't have to worry what you look like. If you are going to come in and no one is going to talk to you, because you know that someone will speak to you that day. … But like I feel like I can come and go home knowing that the kids have had a lovely time, I've had a chance to speak to someone, discuss my concerns, discuss the story that I've been reading, get a new book and then go home and go, “Oh, I've had a nice morning.”

(Focus Group, Area 5b)

Programme theory 8 proposes:

When delivering RfW (Context), the place where the project is delivered (resource) influences people’s willingness and their curiosity to participate by creating a space that feels safe (reasoning) leading to increased participation (outcome).

## Discussion

A salutogenic approach to health promotion postulates that enhancing assets that improve a person’s sense of coherence such as increased sense of self-efficacy and self-acceptance, tolerance of a variety of emotions, perceptions of meaningful connectedness and quality social support, and coping abilities promote positive mental health (Langleland and Vinje 2022; Langeland *et al* 2007; Eriksson and Lindstrom 2010). Within RfW, self-efficacy and connectedness emerged in those who engaged as prominent salutogenic assets that affected participants’ sense of well-being. RfW facilitated an opportunity for reading for pleasure to be considered as a worthy activity and for reading to be experienced socially. Emanating from this, three key interconnected pathways to enhancing an individual’s well-being emerged: affirmative effects of reader identity, enriching connections and celebrating self-care. Facilitation also emerged as a key component in creating a conducive space within the project.

### Affirmative effects of reader identity in the face of emotional hardship

Our findings resonated with the published literature, which highlighted self-identification as a reader or a positive affiliation with reading for pleasure as key factors affecting participation in reading interventions (Garner 2020; Hodge, Robinson, and Davis 2007; Pettersson 2018; Sheldrick Ross 1999; Latchem and Greenhalgh 2014; Thumala Olave 2018; Lang and Brooks 2015). Those who engaged with the evaluation appear to consider reading to be a positive experience, based on their experiences in the past. For these people, RfW provided a social platform to perform and affirm an aspect of their identity, which also bears a positive cultural value in general in British society (Thumala Olave 2018). The positivity associated with reader identity was sometimes contrasted against less positive life experiences, such as bereavement, illness or where participants felt their sense of self had been eroded by duties and responsibilities towards close family. Set against those experiences, some participants described the project spaces as a place to *be* themselves, indicating, as presented in the literature, that participation in the project enabled them to embrace, celebrate and be reassociated with a positive aspect of their identity, which they had considered important (Hammer *et al* 2017; Latchem and Greenhalgh 2014; Lang and Brooks 2015).

RfW appeared to enable a space for participants to harness this positivity, which affected their well-being. Some participants elaborated that making the commitment to engage in an activity that they regarded in a positive light motivated them to act, although in small ways towards self-efficacy, starting from taking steps to leave the house, to self-care, to socialising and helping others. Reflections and conversations within RfW groups presented some of these participants with the opportunity to share and celebrate personal journeys, which further strengthened their sense of efficacy. These hint at the potential project such as RfW could mobilise to ‘activate, emancipate, and increase participants’ perceptions of their resources and potentials that are on the edge of their awareness’ which could improve their well-being (Langeland *et al* 2007, 280).

Previous research on health promotion interventions, drawing from Bourdieu’s theory of practice, have pointed out that closer an individual’s disposition—composite of durable, cognitive, emotional, embodied dispositions—aligns with a proposed course of action, the less effort that is required for that individual to accept or take up the offer and indeed benefit from the offer (Nettleton and Green 2014; Gibson, Pollard, and Moffatt 2021). For a majority of RfW participants who took part in the research, the congruence between previous experiences of reading, which were regarded to be positive, and the offer of RfW appear to enable them to accept the offer of the intervention and to mobilise associated salutogenic assets such as self-efficacy, which seemed to help them face hardships such as emotional hardships.

### Enriching connections

As indicated in the findings, participants’ desire to turn reading into a social experience buttressed participation, which is consistent with the literature (Sheldrick Ross 1999; Lang and Brooks 2015; Pettersson 2018). The analysis showed that, for participants of RfW, coming together as a group facilitated identity-proximity, which instigated connected processes of social identity, emotional sharing and supportive proximity (Pardede, Gausel, and Høie 2020). An awareness of a shared interest in reading and/or a reader identity enabled RfW participants to consider each other as like-minded, which at times traversed intersectional identities and bridged differences in preferences. Terminology such as ‘companions’ or ‘likeminded’ indicated that the single shared interest in reading enabled identity-proximity within RfW groups and aided them to develop a social identity as story lovers. The connectedness, which the participants described, appeared as a compounding effect of positive associations with reading, which was in turn attributed a positive value.

The unique opportunity for in-depth reflection through books and stories added depth and meaning to connectedness. The conversational focus in RfW groups and the possibility to explore a multitude of perspectives on diverse themes via following books appeared to strengthen group identity formation. The diversity of themes and perspectives enabled participants to get to know each other better as well as enhance their understanding of life and the world, instigating multiple pathways for ‘social integration, opportunity for nurturing, reassurance of worth, reliable alliances, and guidance’ (Langeland *et al* 2007, 280). Frequency and regularity of RfW activities further supported social cohesion. Our findings highlight the potential of RfW groups to become a ‘social cure’ for its group members—a connection that begins with a valued shared interest becomes strengthened through meaningful and regular interactions, thus becoming a strong psychological resource for its group members (Jetten *et al* 2017).

### Celebrating self-care

RfW positioned the act of reading for pleasure within a setting of well-being, thus creating an easy passageway for discussing and reflecting on one’s well-being. The engagement with wellbeing consisted of discussing themes such as benefits of reading for pleasure, the joy of connection and engaging in self-care. Escapism and catharsis emerged as key benefits participants experienced through engaging in reading for pleasure. Participants appeared to regard the space within RfW as a space for enjoyment—a space for a cup of tea, company and laughter. Conversations within RfW helped participants to recognise and accept the need for self-care. Further, reading, as indicated above, presented an opportunity to explore a multitude of themes and perspectives, which participants indicated to have affected their perceptions of health and self-care. In addition, within RfW, these conversations were located in an environment that explicitly acknowledged and invited participants to consider their well-being. Combining the space to reflect on well-being and self-care with the potential to explore a multitude of themes through reading, participants appeared to have carved out a space to reflect on quality of life, where they considered life as comprising of multiple components and reflecting on aspects of life that make life meaningful and liveable (Lindström 1992). The conversations within RfW asserted the need to look after oneself, encouraged one another to reflect on opportunities for acts of reading for pleasure to become part of one’s self-care regime and shared resources. While creating an environment where reading for pleasure came to be regarded as an accessible means of self-care, conversations within RfW groups enabled some participants to challenge hindering attitudes towards the practice, such as regarding reading for pleasure as a selfish act. This was particularly relevant for participants who were experiencing emotional hardship.

However, it was indicated that caution is required when explicitly associating the intervention with well-being, as perceptions of stigma associated with poor mental health may inhibit the uptake of the project.

### RfW as a responsive space

Within RfW, the community reading workers played a key role, as they positioned themselves as a ‘dialogue partner, balancing between listening empathetically to participants’ difficulties and taking into account their strengths and resources’ (Langeland *et al* 2007, 280). Further, the setting influenced the level of engagement required of the workers. When reading for pleasure was introduced to pre-existing groups, the worker appeared to take a background role, taking time to understand and cater to needs and group dynamics. Establishing new groups, at the start, revolved around the worker being seen as the locus of the group, where interpersonal needs and dynamics were managed. With time, in some instances, some workers seem retreat to a more withdrawn role whereas on other occasions, the workers remained integral to the group functioning. Workers’ skills and experience to manage interpersonal relationships as well as their knowledge and passion for reading for pleasure appeared as the core strengths that facilitated the reading worker’s role. These findings resonate with the relationship work required of facilitators, which have been highlighted in similar studies relating to artistic practice in general (Reynolds 2018; Bungay, Jensen, and Holt 2023; Belfiore 2022) as well as reading-related activities in particular (Dowrick *et al* 2012).

In addition to interpersonal strengths of community reading workers, RfW’s approach affected its ability to reach and affect its participants. Parting ways from other reading interventions, RfW focused on access, which included widening the understandings of what it means to be a reader. With this approach, RfW embraced a sense of openness, which may have appealed to a group of people, some of who, while regarding reading for pleasure as a valued activity, struggled to read for pleasure. In addition, positioning reading for pleasure within conversational settings, which loosely alluded to well-being and mental health appeared to have marked RfW as distinct from bibliotherapy, thus veering away from potential stigma that may be associated with interventions explicitly associated with mental health. This disassociation from bibliotherapy may have influenced participants’ notions of the project’s accessibility.

### Strengths/limitations

The explanatory and iterative nature of realist approaches enabled the evaluation to attend to diverse, complex components and diverse contexts of the project. Further, early involvement with the RfW project facilitated a grounded approach to theory elicitation and refinement.

While the proposition for realist interviews is that sampling is theory based, the extent to which we could influence sampling was limited, as we accessed participants through gatekeepers. A key consequence of this is that we were limited in our ability to explore the perspectives of those who did not engage in the research and those who did not engage in the project. Therefore, we are limited in our ability to explore the relevance of the finding to those who engaged with the project but not the evaluation and for those who did not engage with RfW, for whom the causal associations may be different.

## Conclusion

This paper presented the findings of a realist evaluation of the RfW project. The findings of this research indicate the primacy of positive associations with reading for pleasure for interventions such as RfW to trigger pathways to well-being. For the participants, positive association with reading for pleasure appeared to have helped for RfW to be seen as an activity with positive value, therefore was positioned within a locus of positivity. Within the project, this imbued positivity instigated salutogenic assets related to self-efficacy, well-being and self-care. In addition, the opportunity to engage in reading for pleasure as a social experience engendered multiple resources that appeared to increase an individual’s ability to cope with life’s stressors, particularly through increasing sense of connection and belonging to the RfW group and providing a platform where participants collectively reflected on practices of self-care, notions of health and well-being, and quality of life. This finding highlights the importance of context for health promotion interventions such as RfW for it is the interaction between the context of positive associations and the resources that were introduced through RfW that triggered pathways to harnessing salutogenic assets, which in turn positively affected participants’ sense of well-being. Community reading workers played a key role in the facilitation of this process, mobilising their skills, knowledge, dispositions and passion for reading to form effective connections with the participants at an individual level, addressing their needs and facilitating enhancing environments to foster group connections.

In some pilot areas, RfW continues to be funded while in some areas, activities inspired by RfW have been incorporated into library service. RfW has also been rolled out in other local authority areas.

## Data Availability

No data are available.

## Footnotes

In the following sections, references to reading includes these multiple ways of enjoying stories.
